# CMTM5 is downregulated and suppresses tumour growth in hepatocellular carcinoma through regulating PI3K-AKT signalling

**DOI:** 10.1186/s12935-017-0485-8

**Published:** 2017-11-29

**Authors:** Gang Xu, Chengxue Dang

**Affiliations:** Department of Surgical Oncology, The First Affiliated Hospital Xi’an Jiaotong University, No. 277 West Yanta Road, Xi’an, 710061 Shaanxi People’s Republic of China

**Keywords:** CMTM5, Hepatocellular carcinoma, Tumour suppressor, PI3K-AKT

## Abstract

**Background:**

Human chemokine like factor (CKLF)-like MAL and related proteins for vesicle trafficking transmembrane, domain-containing member 5 (CMTM5) has been shown to involved and may function as a tumour suppressor in tumorigenesis. The current study aimed to investigate the expression and function of CMTM5 in human hepatocellular carcinoma (HCC).

**Methods:**

CMTM5 expression was examined by immunohistochemistry, and its clinical significance was analysed in 76 HCC specimens. The role and molecular mechanisms of CMTM5 in cell proliferation, apoptosis and invasion were examined in vitro and in vivo.

**Results:**

CMTM5 expression was significantly downregulated in HCC tissues as well as cell lines. The expression of CMTM5 was absent in 77.6% of HCC tissues compared with 3.9% in normal liver tissues. Low CMTM5 expression was significantly correlated with poor overall survival in patients with HCC (*P* = 0.009). Restoring CMTM5 expression in Huh7 cells significantly inhibited cell growth, promoted cell apoptosis, and reduced cell metastatic and invasion ability compared with mock transfected cells in vitro. Overexpression of CMTM5 also suppressed xenograft tumour growth in vivo in a HCC xenograft model. Reduced cell growth and metastasis ability mediated by CMTM5 overexpression was associated with downregulation of PI3K/AKT and its downstream Bcl2, cyclinD1, cyclinE, MMP2 and MMP9 expressions, and an upregulation of p21, Bax, Bad, cleaved caspase3 expressions.

**Conclusions:**

Our data suggest that CMTM5 might function as a tumour suppressor in human HCC, and represent a valuable potential therapeutic target for HCC.

**Electronic supplementary material:**

The online version of this article (10.1186/s12935-017-0485-8) contains supplementary material, which is available to authorized users.

## Background

Liver cancer is a common malignant and the fourth leading cause of cancer deaths in China [1]. It is estimated 466,100 new cases of liver cancer in 2015 in China, with 422,100 new deaths due to this disease [1]. Hepatocellular carcinoma (HCC) is the most common type and accounts for 70–85%of liver cancer [2]. People with early-stage HCC can be treated with surgery, either removing part of the liver or transplantation, with 5-year survival rates that exceed 70%. Most people, however, cannot have surgical treatment and so the overall 5-year survival is about 15% [3]. Therefore, it is important to understand the molecular mechanisms involved in HCC carcinogenesis, and find novel approaches that can effectively inhibit HCC cell growth and metastasis.

Human chemokine-like factor (CKLF)-like MARVEL transmembrane domain-containing family (CMTM) is a novel family consisting of nine genes, CKLF and CMTMI to CMTM8. CMTMs, linking classical chemokines and the transmembrane-4 superfamily (TM4SF), play important roles in immune system, male reproductive system and carcinogenesis [4, 5]. CMTM5 was firstly reported by in 2003, and located at 14q11.2 [4]. It is structurally similar to the TM4SF which can by collecting a variety of other molecules (such as AKT, EGFR) form four cross membrane protein network, thus mediated cell proliferation, apoptosis, migration, adhesion, differentiation, and closely related to tumour formation [4, 6, 7]. It has been reported that CMTM5 acted as a tumour-suppressor gene and was specifically down-regulated in many human cancers, such as cervical carcinoma [8], pancreatic cancer [9], myeloid leukemia [10], ovarian cancer [11], oral squamous cell carcinoma (OSCC) [12] and prostate cancer [13]. However, the clinical significance and function of CMTM5 in HCC remains unclear.

In this study, we aimed to detect the expression patterns of CMTM5 and their correlations with clinicopathological characteristics in HCC patients. Furthermore, we explored the effect and mechanisms of CMTM5 on cell growth and metastasis based on HCC cell line in vitro and in vivo.

## Methods

### Tissue samples and cell culture

The study was approved by the ethics committee of Chinese PLA General Hospital (CHN-PLAGH-ZL-002), and informed consent was obtained from each patient according to the committee’s regulations. Tissue samples collected from 76 patients who underwent curative liver resection at the Department of Gastroenterology and Hepatology at the Chinese PLA General Hospital between 2007 and 2010 were used for CMTM5 immunohistochemistry detection. TNM classification was determined according to the 2002 International Union Against Cancer guidelines. Tumour differentiation was graded according to the Edmondson-Steiner classification. Clinical characteristics of all patients are shown in Table 1. The time to overall survival (OS) time were defined as the interval between surgical treatment and death. HCC cell lines, Huh7, Hep3B, HepG2 and SMMC-7721, and human normal liver cell line L02 were maintained in Dulbecco’s modified Eagle’s medium (DMEM; Gibco, Grand Island, NY, USA) supplemented with 10% foetal bovine serum (FBS) at 37 °C with 5% CO_2_ humidified atmosphere.

**Table 1:** Correlation between CMTM 5 expression and clinicopathological variables of 76 HCC patients

Variables	Cases (76)	CMTM5 expression		*P* value
		High (17)	Low (59)	
Age (years)				
≤ 50	47	12 (70.6%)	35 (59.3%)	0.572
> 50	29	5 (29.4%)	24 (40.7%)	
Gender				
Male	57	11 (64.7%)	46 (77.9%)	0.266
Female	19	6 (35.3%)	13 (22.1%)	
AFP (ng/mL)				
≤ 20	35	7 (41.2%)	28 (47.5%)	0.647
> 20	41	10 (58.8%)	31 (52.5%)	
ALT (U/mL)				
≤ 75	40	10 (58.8%)	30 (50.8%)	0.564
> 75	36	7 (41.2%)	29 (49.2%)	
GGT (U/mL)				
≤ 45	43	11 (64.7%)	32 (54.2%)	0.443
> 45	33	6 (35.3%)	27 (45.8%)	
Tumor size (diameter), cm				
≤ 5	34	10 (58.8%)	24 (40.7%)	0.185
> 5	42	7 (41.2%)	35 (59.3%)	
Tumor multiplicity				
Single	53	12 (70.6%)	41 (69.5%)	0.931
Multiple	23	5 (29.4%)	18 (30.5%)	
Vascular invasion				
Yes	20	5 (29.4%)	15 (25.4%)	0.742
No	56	12 (70.6%)	44 (74.6%)	
Tumor differentiation				
I–II	57	16 (94.1%)	41 (69.5%)	0.039*
III–IV	19	1 (5.9%)	18 (30.5%)	
TNM stage				
I	41	14 (82.4%)	27 (45.8%)	0.028*
II	25	2 (11.7%)	23 (38.9%)	
III	10	1 (5.9%)	9 (15.3%)	

* *P* < 0.05

### Immunohistochemistry and evaluation

CMTM5 expression was detected by streptavidin peroxidase conjugated method. Briefly, formalin fixed, paraffin-embedded tissues were cut into 4-μm-thick sections, and treated with 3% H_2_O_2_ methanol at room temperature for 10 min. Sections were then incubated in 5% goat antiserum for 15 min at 37 °C. After rinsing with PBS, the sections were incubated with rabbit polyclonal to human CMTM5 antibody (ab187980; Abcam, Cambridge, MA, USA), mouse monoclonal to human PI3K p85 (ab86714, Abcam), rabbit anti human phospho-Akt (Ser473) (4060, 1:100, CST) at 4 °C overnight. The sections were then washed and incubated with horseradish peroxidase (HRP)-conjugated goat anti-rabbit IgG (ab205718, 1:5000, Abcam) for 30 min followed by further incubation with the streptavidin–horseradish peroxidase complex. The slides were washed and visualized by adding the substrate diaminobenzidine tetrahydrochloride and then counterstained with haematoxylin.

CMTM5 signal was calculated based on the intensity and the percentage of positively stained cells. The intensity of the staining was scored as follows: 0 (no staining), 1 (weak staining), 2 (moderate staining) and 3 (strong staining), respectively. The staining extent was scored from 0 to 3 based on the percentage of positive cells (0: < 10%; 1: 10–25%; 2: 25–50% and 3: > 50%). The final CMTM5 score was calculated using the percentage of positive cells × staining intensity, ranging between 0 and 9. For all cases, total score of ≥ 4 was defined as high expression, and score of 0–3 was defined as low expression.

### Cell infection

Full-length CMTM5 cDNA (Sino Biological, Beijing, China) was cloned into pLenti6.3-IRES2-EGFP lentiviral vector (Invitrogen, Carlsbad, CA, USA) to generate pLenti6.3-CMTM5-IRES2-EGFP. pLenti6.3-IRES2-EGFP lentivirus was used as a negative control. Huh7 or Hep3B cells were infected with the recombinant lentivirus carrying CMTM5 at a multiplicity of infection (MOI) of 10. The EGFP signal was observed by fluorescence microscopy. Stably transfected clones were validated by qRT-PCR and western blotting.

### Real-time quantitative RT-PCR

Total RNA was extracted using TRIzol (Invitrogen) and reverse transcribed using M-MLV reverse transcriptase (Life Technologies Inc., Gaithersburg, MD, USA). Primers used in this study were shown in Additional file 1: Table S1. Data were analysed using the comparative 2^−ΔΔCt^ method.

### Western blotting

Total protein was extracted, separated on SDS-PAGE and transferred to a polyvinylidene fluoride membrane. Membrane was blocked with 5% non-fat milk in TBS and then incubated with antibodies against CMTM5 (ab96077, Abcam), AKT (4691, CST), pAKT (4051, CST), PI3K (ab86714, Abcam), p21 (2946, CST), CyclinD1 (2978, CST), CyclinE (4129, CST), Bcl2 (ab117115, Abcam), Bax (AB32503, Abcam), Bad (ab32445, Abcam), cleaved caspase 3 (9661, CST), MMP2 (13132, CST), MMP9 (13667, CST) overnight at 4 °C. HRP-conjugated IgG antibody were treated at 37 °C for 1 h. The bands were then visualized using BioImaging Systems (UVP Inc., Upland, CA, USA).

### PI3K activity assay

The PI3K activity was measured using an enzyme-linked immunosorbent assay kit (Echelon Biosciences, Salt Lake City, UT, USA) according to the manufacturer’s instructions. Briefly, cell lysate was incubated with an immobilized anti-p85α antibody overnight. The immunoprecipitates were then incubated with a reaction mixture containing phosphatidylinositol (PtdIns)-4,5-P2 substrate and ATP. The reaction mixtures were first incubated with an antibody to PtdIns-3,4,5-P3 and then added to the PtdIns-3,4,5-P3-coated microplate for competitive binding. Peroxidase-linked secondary antibody and colorimetric detection were used to detect anti-PtdIns-3,4,5-P3 binding to the plate. The colorimetric signal was inversely proportional to the amount of PtdIns-3,4,5-P3 produced by activated PI3K.

### Cell proliferation assay

Cellular proliferation was evaluated using Cell Counting Kit-8 (CCK-8; Beyotime Biotechnology, Shanghai, China) according to the manufacturer’s instructions. Briefly, 5 × 10^3^ cells infected with CMTM5 or mock were seeded into 96-well plates and cultured for the indicated time. Optical density values were read using a microplate reader (Bio-Tek, Winooski, VT, USA) at a wavelength of 450 nm. In some experiments, PI3K inhibitor LY294002 (10 μM, Selleck, Houston, TX, USA) was added in the culture, DMSO was used as vehicle control.

### Cell apoptosis assay

Cell apoptosis was evaluated by flow cytometry using an Annexin V-FITC Apoptosis Detection Kit (Beyotime). Briefly, 1 × 10^5^ cells infected with CMTM5 or mock were washed twice with PBS, and resuspended in 500-μL binding buffer with 10-μL Annexin V-FITC and 5-μL propidium iodide in the dark for 15 min. Data were acquired immediately by flow cytometry (BD FACSCalibur, San Diego, CA, USA) and analysed using FlowJo software (FlowJo, Ashland, OR, USA).

### Cell migration and invasion assay

The migration assay was performed in a 24-well transwells (8-μm pore size; Minipore, Billerica, MA, USA), according to the manufacturer’s instruction. Briefly, 2 × 10^5^ cells in 200 μL DMEM containing with 1% FBS were added to the upper chamber, 600 μL of DMEM containing 10% FBS was added in the lower chamber. 24 h after incubation, nonmigrated cells remaining in the upper chamber were removed, and the migrated cells on lower surface of the membrane were counted through fixing with 4% paraformaldehyde and staining with Giemsa. For invasion assay, the chamber was pre-coated with Matrigel (BD Biosciences, Franklin Lakes, NJ, USA).

### In vivo tumour xenograft model

Male BALB/c nu/nu mice (6–8 week-old) were purchased from Charles River Laboratories, Beijing, China) and housed under specific pathogen-free conditions. All animals received humane care according to the criteria outlined in the Guide for the Care and Use of Laboratory Animals prepared by the National Academy of Sciences and published by the National Institutes of Health (NIH publication 86–23 revised 1985). Animal study protocols were approved by the Research Ethics Committee of Chinese PLA General Hospital. 5 × 10^6^ Huh7-CMTM5 cells or mock transfected Huh7 cells were injected subcutaneously into the flank region of nude mice to establish xenograft HCC model (n = 6 per group). Tumour growth was monitored every 3 days for 4 weeks, tumour volume was measured with fine digital callipers and calculated using the following formula: 0.5 × width^2^ × length. Mice were sacrificed 4 weeks later and tumours were weighed and photographed.

### Statistical analysis

Statistical analyses were performed using the Statistical Package for the Social Sciences software 19.0 (SPSS, Chicago, IL, USA). The relationship between CMTM5 expression and clinicopathological characteristics were analysed by Chi square test. Survival curves were plotted using the Kaplan–Meier method and compared using the log-rank test. Cox proportional hazard regression model was used for univariate and multivariate analyses. The other results were assessed using Student’s *t* test or One-Way ANOVA with a post hoc analysis. Data are presented as mean ± SD. *P* < 0.05 was considered to be statistically significant.

## Results

### CMTM5 expression is downregulated in HCC tissues and cell lines

**Fig. 1:**
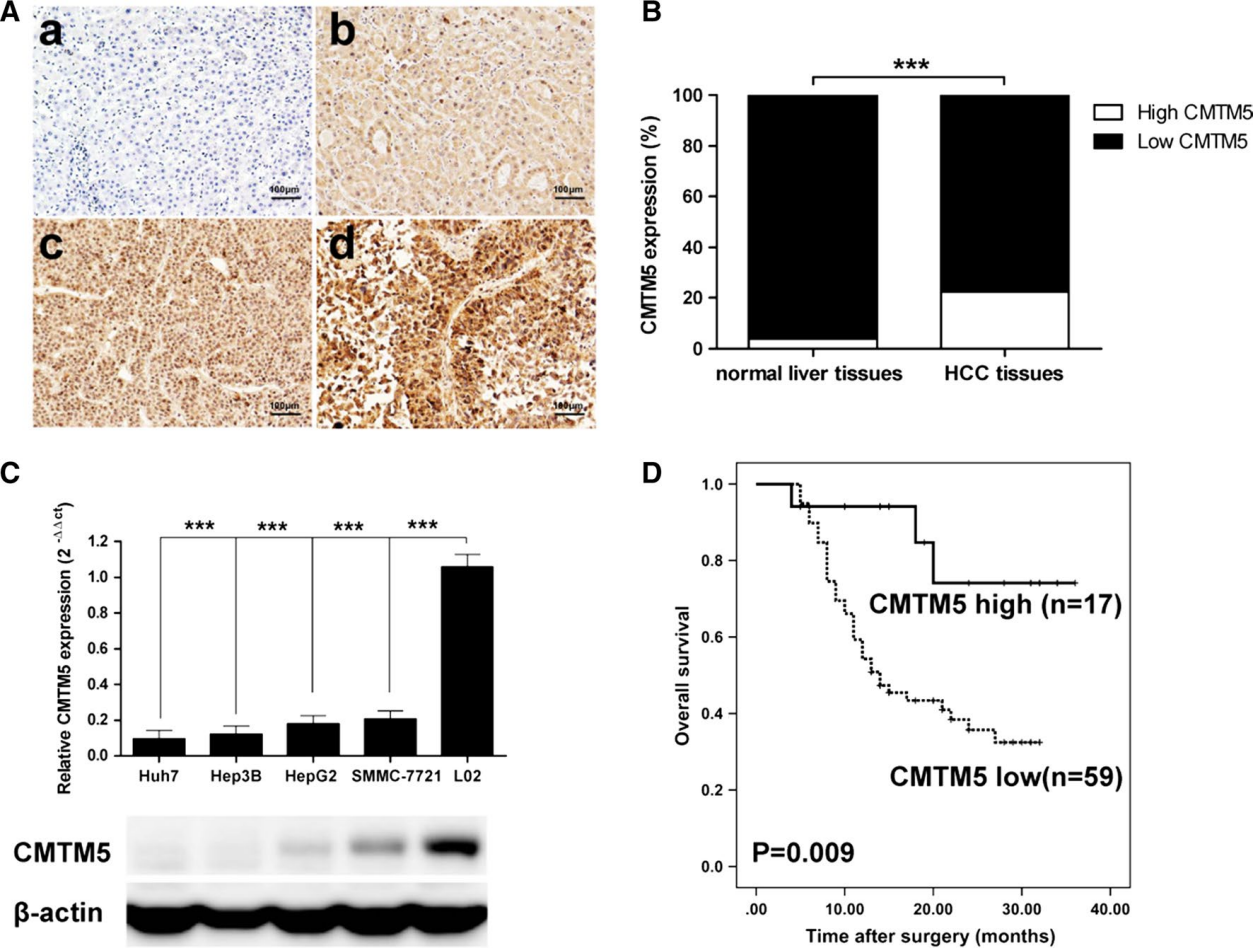
CMTM5 expression is downregulated in HCC tissues and cell lines. **A** Representative photos of CMTM5 staining in HCC and adjacent liver tissues (a, low, b, high, CMTM5 expression in HCC; c, low, d, high, CMTM5 expression in normal liver tissues; ×400). **B** The ratio of high CMTM5 expression between HCC tissues and normal live tissues, ***P < 0.001, high CMTM5 expression of HCC tissues vs. high CMTM5 expression of normal live tissues (χ^2^ test). **C** qRT-PCR and western blotting analysis of CMTM5 expression in HCC cell lines Huh7, Hep3B, HepG2, SMMC-7721 and in normal liver cell line L02. Data are shown as mean ± SD from three independent experiments, ***P < 0.001, compared with L02 group (One-Way ANOVA with Dunnett’s test). **D** Kaplan–Meier survival curves for overall survival according to CMTM5 expression in tumor tissues from 76 HCC patients. The P value was calculated using the log-rank test

The CMTM5 expression was examined by immunohistochemistry staining in 76 paired tumour and adjacent normal liver tissues. As shown in Fig. 1A, CMTM5 expression was observed predominantly in the cytoplasm. Most of the normal liver tissues were scored strong positive (43/76, 56.6%) or moderate positive staining (30/76, 39.5%), only 3 cases were weakly staining (3/76, 3.9%). However, in 76 HCC specimens, CMTM5 was either negative (39/76, 51.3%) or weakly (20/76, 26.3%) expressed, with only a few of moderate positive staining (17/76, 22.4%), and no strong positive staining. There was a significant difference in the ratio of high CMTM5 expression between HCC tissues and normal live tissues (*P* < 0.001, Fig. 1B). To further confirm the above results, we next examined the expression of CMTM5 in four HCC cell lines (Huh7, Hep3B, HepG2 and SMMC-7721) and one normal liver cell line (L02). The results demonstrated that the mRNA and protein expression of CMTM5 were almost undetectable in four human HCC cell lines, but CMTM5 was highly expressed in L02 cells (*P* < 0.001, Fig. 1C). Taken together, our results suggest that CMTM5 expression is downregulated in HCC tissues and cell lines.

### Low CMTM5 expression is associated with poor prognosis in HCC

To determine the clinical significance of CMTM5 expression in HCC, we evaluated the relationships between CMTM5 expression and clinicopathological characteristics. As shown in Table 1. No statistically significant associations were observed between CMTM5 expression and various clinicopathological features, such as age, gender, AFP level, ALT level, GGT level, tumour size, tumour multiplicity, as well as vascular invasion. However, there was a significantly correlation between the CMTM5 expression and tumour differentiation (*P* = 0.039) and TNM stage (*P* = 0.028). In addition, the survival data from 76 patients with HCC was also assessed. As shown in Fig. 1D, the overall survival of patients with a high CMTM5 expression was significantly longer than the survival of those with low CMTM5 expression (*P* = 0.009). Furthermore, As shown in Table 2, the univariate analyses showed that tumour size (*P* = 0.009), vascular invasion (*P* = 0.014), tumour differentiation (*P* = 0.037), TNM stage (*P* = 0.003) and CMTM5 expression (*P* = 0.008) were associated with overall survival. Multivariate analysis indicated that tumour size (*P* = 0.011), vascular invasion (*P* = 0.033), TNM stage (*P* = 0.009) and CMTM5 expression (*P* = 0.013) were independent prognostic factors for overall survival in patients with HCC (Table 2).

**Table 2:** Univariate and multivariate analyses of factors associated with overall survival of HCC patients

Factors	Univariate	Multivariate		
	*P*	Hazard ratio	95% CI	*P*
Age, years (≤ 50 vs > 50)	0.611			
Gender (male vs female)	0.423			
AFP (ng/mL) (> 20 vs ≤ 20)	0.249			
ALT (U/mL) (> 75 vs ≤ 75)	0.598			
GGT (U/mL) (> 45 vs ≤ 45)	0.314			
Tumor size (cm) (> 5 vs ≤ 5)	0.009*	2.044	1.019–3.332	0.011*
Tumor multiplicity (multiple vs single)	0.134			
Vascular invasion (yes vs no)	0.014*	2.218	0.891–3.013	0.033*
Tumor differentiation (I–II vs III–IV)	0.037*	1.877	1.035–2.821	0.098
TNM stage (I–II vs III)	0.003*	3.691	0.987–5.401	0.009*
CMTM5 expression (high vs low)	0.008*	3.087	1.012–4.392	0.013*

* *P* < 0.05

### Overexpression of CMTM5 inhibits HCC cell growth and metastasis in vitro

**Fig. 2:**
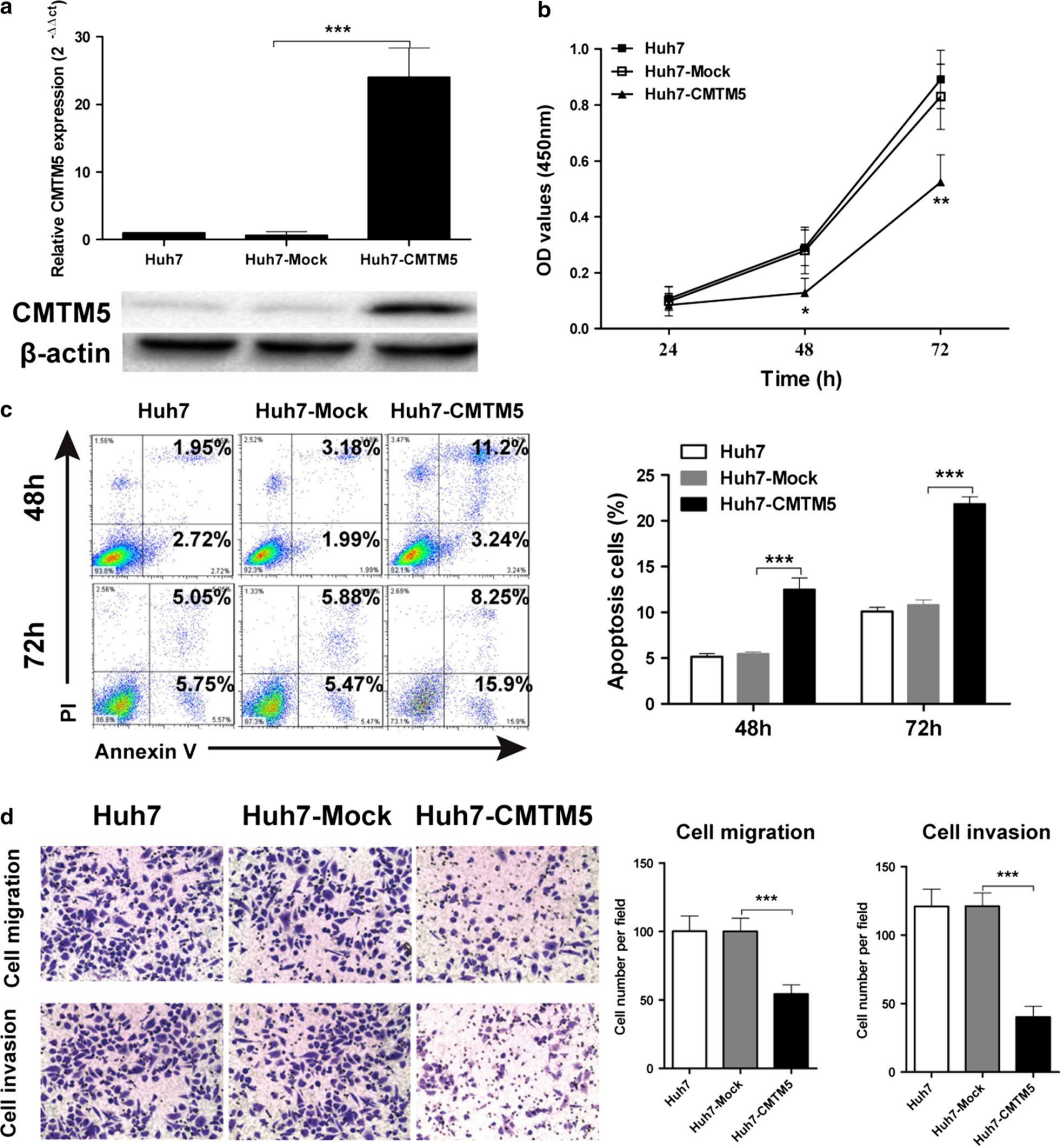
Overexpression of CMTM5 inhibits HCC cell growth and metastasis in vitro. **a** Lentivirus vector pLenti6.3-CMTM5-IRES-EGFP and the mock control lentivirus were transfected into Huh7 cells. Transfection efficiency was confirmed by qRT-PCR and western blotting. **b** Cell growth was assessed by time course CCK-8 assay. **c** Cell apoptosis was determined by flow cytometry using the Annexin V/PI staining. **d** Cell metastatic and invasion was assessed by Transwell assay. Data are shown as mean ± SD from three independent experiments. *P < 0.05, **P < 0.01, ***P < 0.001, compared with Huh7-Mock group (One-Way ANOVA with Dunnett’s test)

To examine the function of CMTM5 in HCC, lentivirus vector pLenti6.3-CMTM5-IRES-EGFP and the mock control lentivirus were transfected into Huh7 cells. The restoration of CMTM5 mRNA and protein in Huh7 cells was confirmed by qRT-PCR and western blotting (*P* < 0.001, Fig. 2a). Cell growth was examined by CCK-8 assay, as shown in Fig. 2b, the proliferation of Huh7-CMTM5 cells was significantly decreased compared to mock control cells at the time point 48 and 72 h (*P* < 0.05 and *P* < 0.01, respectively). transfection. The effect of CMTM5 on cell apoptosis was determined by flow cytometry using the Annexin V/PI staining. Data showed that the apoptosis rate of CMTM5 infected Huh7 cells were significantly higher than the mock control cells at the time point 48 and 72 h after transfection (*P* < 0.001, respectively, Fig. 2c). Additionally, using Transwell assay, we found that restoration of CMTM5 significantly reduced cell metastatic and invasion ability compared with mock transfected cells (*P* < 0.001, respectively, Fig. 2d). The effect of CMTM5 on HCC growth and metastasis in vitro was confirmed by using another HCC cell lines Hep3B (Additional file 2: Figure S1A–F). Taken together, our results suggest that restoration of CMTM5 inhibits cell growth and metastasis of Huh7 cells in vitro.

### Overexpression of CMTM5 suppresses xenograft tumour growth in vivo

**Fig. 3:**
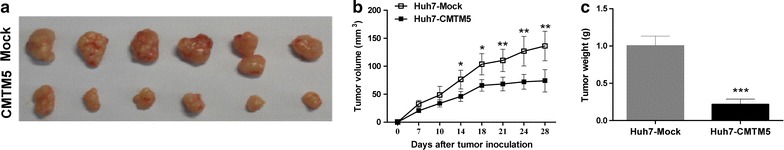
CMTM5 suppresses tumor growth in vivo. Huh7-CMTM5 cells and mock transfected Huh7 cells were injected subcutaneous into the flank region of nude mice. Tumor volume was calculated every 3 days after implantation. **a** Photographs showed the CMTM5 or mock transfected xenografts at the end of the experiment. **b** The tumor growth curve and **c** tumor weight of two groups were shown. Data are shown as mean ± SD from three independent experiments. *P < 0.05, **P < 0.01, compared with Huh7-Mock group (Student’s *t* test)

To elucidate the effect of CMTM5 on HCC growth in vivo, we injected Huh7-CMTM5 cells and mock transfected Huh7 cells into nude mice to establish xenograft HCC model. As shown in Fig. 3a, b, 4 weeks after tumour injection, CMTM5 transfected xenografts had a much slower growth curve compared with mock transfected xenografts. In addition, tumour weight of CMTM5 transfected xenografts was significantly smaller than that of controls (Fig. 3c). These results indicated that overexpression of CMTM5 suppresses xenograft tumour growth in vivo.

### CMTM5 inhibits tumour growth and metastasis through regulating PI3K-AKT signalling

**Fig. 4:**
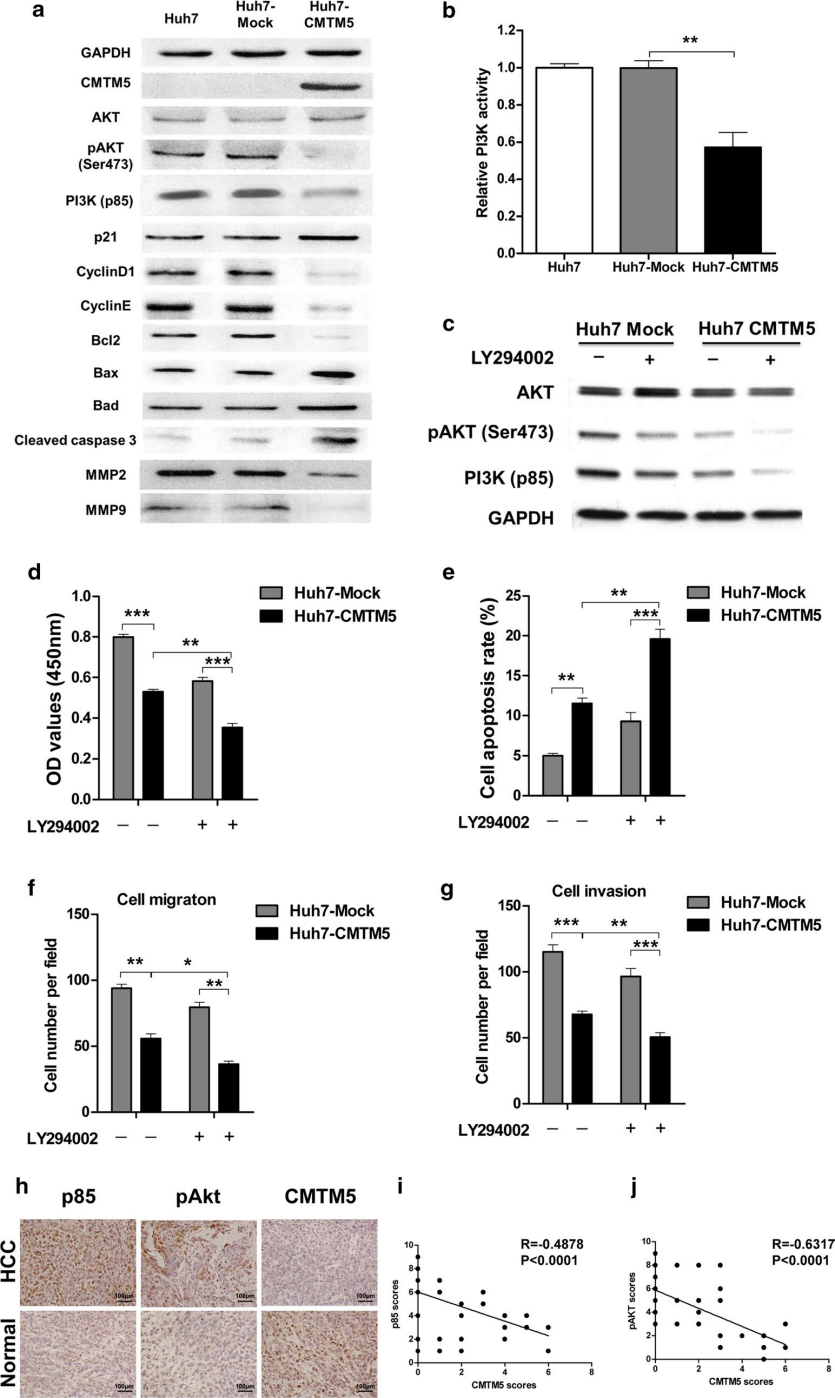
CMTM5 inhibits tumour growth and metastasis through regulating PI3K-AKT signalling. **a** The related proteins of PI3K/Akt pathway, including AKT, pAKT, PI3K p85 subunit, p21, cyclinD1, cyclinE, Bcl2, Bax, Bad, cleaved caspase 3, MMP2 and MMP9 in CMTM5 overexpressed Huh7 cells were examined by western blotting. **b** The relative kinase activity of PI3K in CMTM5 overexpressed Huh7 cells were examined by PI3K kinase ELISA assay. **c** The proteins levels of AKT, pAKT and P85 in Huh7-CMTM5 cells after treatment of PI3K inhibitor LY294002 (10 μM). **d**–**g** Huh7-CMTM5 cells were treated with or without LY294002, Cell growth (**d**), cell apoptosis (**e**) and cell metastatic and invasion (**f**, **g**) were examined. **h**–**j** The expression of PI3K p85 and pAKT (Ser473) in 76 paired tumour and adjacent normal liver tissues were examined by IHC staining and scored from 0 to 9 as described in the method. The left plots (**h**) were representative results of IHC staining against indicated proteins. The right plots were the statistical analysis of the correlation of CMTM5 expression with p85 (**i**) and pAKT (**j**) in HCC tissues (Spearman correlation test). Data are shown as mean ± SD from three independent experiments. *P < 0.05, **P < 0.01, ***P < 0.001, One-Way ANOVA with Dunnett’s test

To investigate the molecular mechanisms by which CMTM5 affects HCC growth and invasion, we explored the related mRNA and proteins of PI3K/AKT pathway, which is a prototypic survival pathway implicated in HCC carcinogenesis. As shown in Fig. 4a and Additional file 2: Figure S1G–P, there were significant decreasing of PI3K p85, pAKT (Ser473) and its downstream Bcl2, cyclinD1, cyclinE, MMP2 and MMP9 in CMTM5-transfected cells, while the increasing of p21, Bax, Bad, cleaved caspase3 compared with the mock-transfected cells. In addition, restoration of CMTM5 significantly inhibited the kinase activity of PI3K in Huh-7 cells (*P* < 0.01, Fig. 4b). To further clarify that PI3K/AKT is responsible for suppression of tumour growth in CMTM5-transfected human HCC cells, we used PI3K/AKT inhibitor LY294002 to examine the effect of AKT inhibition on CMTM5-induced tumour growth suppression (Fig. 4c). The results showed that interference of PI3K/AKT pathway by LY294002 suppressed cell growth, promoted cell apoptosis, and reduced cell metastatic and invasion ability in mock transfected Huh7 cells. However, in Huh7-CMTM5 cells, treatment of LY294002 enhanced the inhibition of cell growth by CMTM5 (Fig. 4d), further promoted the CMTM5 induced cell apoptosis (Fig. 4e), and synergistically inhibited the CMTM5 overexpression-reduced cell metastasis and invasion (Fig. 4f, g). These results suggest that CMTM5 inhibited tumour growth and metastasis mainly through inactivation of PI3K-AKT signaling.

### CMTM5 is negatively associated with the expression of PI3K and pAKT in HCC

To investigate the potential clinical significance of CMTM5 and PI3K/AKT and pathways in human HCC. tissue sections from clinical HCC patients were applied to immunohistochemical (IHC) staining against PI3K p85 and pAKT (Ser473). The results showed that strong staining of p85 and pAKT (Ser473) was observed in HCC tumor tissues (Fig. 4h). In contrast, very low CMTM5 staining was observed in HCC tumor tissues (Fig. 4h). In addition, p85 (R = − 0.4878, P ˂ 0.0001) and pAKT (R = − 0.6317, P ˂ 0.0001) expressions were negatively related with CMTM5 expression in human HCC tissues (Fig. 4i, j). Taken together, these results suggest that CMTM5 might down-regulate PI3K/AKT pathway, leading to inhibition of HCC development.

## Discussion

CMTM5, a member of the human CKLF-like MARVEL transmembrane domain containing family, has recently been recognized as a novel tumour suppressor, as evidenced by reduced or silenced expression in most cancers including cervical carcinoma [8], pancreatic cancer [9], myeloid leukemia [10], ovarian cancer [11], OSCC [12] and prostate cancer [13]. However, the clinical significance and biological role of CMTM5 in HCC remain largely unknown.

Using immunohistochemistry, we found for the first time that CMTM5 expression was significantly reduced in 59 out of 76 (77.6%) HCC tissues, but only lost in three normal liver tissues. It was reported that CMTM5 was decreased markedly in 64.1% (41/64) of prostate cancer tissues [13], and was absent in 30% (24/80) of ovarian cancer tissues [11], suggesting that the loss of CMTM5 is common in carcinoma tissues. Previous studies have shown that CMTM5 is silenced by methylation in many carcinoma cell lines, including HCC [6]. In this study, we confirmed that CMTM5 was also undetectable in Huh7, Hep3B, HepG2, and expressed weakly in SMMC-7721 cells. Since CMTM5 promoter methylation was the main mechanism of gene silencing, the promoter methylation status of CMTM5 should be examined in HCC cell lines in the future study. Taken together, our results demonstrate that CMTM5 expression is frequently lost or reduced in HCC tissues and cell lines.

Herein, we also showed detailed correlations between the CMTM5 expression, clinicopathological features and the prognosis of HCC patients. Low CMTM5 expression was significantly correlated with poor tumour differentiation and advanced TNM stage. Reduced CMTM5 expression were a predictor of worsened survival in HCC patients. In addition, low CMTM5 expression was an independent prognostic factor for poor overall survival. In the previous study, Li et al. found that CMTM5 expression was associated with tumour differentiation and CA125 levels in ovarian cancer patients [11]. Its expression, however, had no significant correlation with the prognosis of patients with ovarian cancer [11]. Collectively, our findings indicate that CMTM5 may function as a tumour suppressor in HCC carcinogenesis.

Previous studies have shown that the restoration of CMTM5 strongly suppresses tumour cell growth and migration in several cancer cells [8, 12, 13]. Overexpression of CMTM5 in cervical carcinoma cell lines Hela and SiHa could induce cell apoptosis mainly through caspase-dependent pathway [8]. Zhang et al., showed that CMTM5 levels could be upregulated by demethylating agent 5-aza-2′-deoxycytidine, ectopic expression of CMTM5 strongly inhibited cell proliferation and migration and induced apoptosis in OSCC cell lines CAL27 and GNM [12]. Moreover, ectopic expression of CMTM5 in prostate cancer cell line DU145 led to significant inhibition of cell proliferation and migration [13]. In this study, we also found that restoring CMTM5 expression in Huh7 cells not only significantly suppressed tumour proliferation, promoted cell apoptosis, but also inhibited cell metastasis and invasion in vitro. Additionally, overexpression of CMTM5 also significantly suppressed xenograft tumour growth in vivo. Our findings demonstrate that CMTM5 might act as a putative tumour suppressor in HCC. However, the molecular mechanisms of CMTM5 inhibits HCC cell growth and metastasis are poorly characterized.

In the previous studies, Guo et al. reported that restoration of CMTM5 inducesd pancreatic cancer cells apoptosis with activation of caspase 3, 8 and 9 [9]. CMTM5 could also induced apoptosis of cervical carcinoma cells through disruption of mitochondrial transmembrane potential, release of cytochrome c, activation of caspase3 and cleavage of PARP [8]. Studies on other CMTM family proteins will uncover their roles and mechanisms in cancer. For instance, CMTM7, another member of the CMTM family, has been shown to inhibit cancer cell growth by inhibiting oncogenic EGFR signalling and the Akt signalling pathway [14, 15]. Knockdown of CMTM3 promoted metastasis of gastric cancer via the STAT3/Twist1/EMT signalling pathway [16]. Moreover, down-regulation of CMTM8 induced epithelial-to-mesenchymal transition-like changes via c-MET/extracellular signal-regulated kinase (ERK) signalling [17]. It has been shown that PI3K/Akt signalling pathway components are frequently altered in human cancers and related to numerous cellular processes of cell growth, survival and invasion [18]. Thus, we supposed that the mechanisms of CMTM5 acting on HCC cells might be involved in PI3K-AKT signalling. Recently, it has been reported that ectopic expression of CMTM5 in prostate cancer cell decreased Akt activity [13]. In the present study, restoring of EMP3 reduced the expression and the activity of p85 subunit of PI3K, which led to inactivation of Akt. In addition, treatment of LY294002 further enhanced the inhibition of cell growth and metastasis by CMTM5. We also observed significantly negative-correlation between CMTM5 levels and the expression of PI3K and pAKT in HCC tissues, suggesting that CMTM5 may inhibit HCC growth and metastasis through regulating PI3K-AKT signalling. However, The mechanisms by which CMTM5 suppresses both PI3K/Akt pathway need further investigation.

## Conclusions

In conclusion, our study provided the first evidence of the reduced expression and the tumor suppressor role of CMTM5 in HCC through regulating PI3K-AKT signalling. CMTM5 might serve as a valuable prognostic marker and potential therapeutic target for HCC.

## Additional files

**Additional file 1: Table S1.** Primers used in this study.

**Additional file 2: Figure S1.** Overexpression of CMTM5 inhibits HCC cell growth and metastasis in Hep3B cells. (A) Lentivirus vector pLenti6.3-CMTM5-IRES-EGFP and the mock control lentivirus were transfected into Hep3B cells. Transfection efficiency was confirmed by qRT-PCR and western blotting. (B) Cell growth was assessed by time course CCK-8 assay. (C) Cell apoptosis was determined by flow cytomety using the Annexin V/PI staining. (D–F) Cell metastatic and invasion was assessed by Transwell assay. (G–P) The mRNA levels of PI3K/Akt pathway, including AKT, PI3K p85, p21, CyclinD1, CyclinE, Bcl2, Bax, Bad, MMP2 and MMP9 in CMTM5 overexpressed Huh7 cells were examined by real-time quantitative RT-PCR. Data are shown as mean ± SD from three independent experiments. *P < 0.05, **P < 0.01, ***P < 0.001, compared with Huh7-Mock group (One-Way ANOVA with Dunnett’s test).

## Abbreviations

CMTM5: chemokine-like factor (CKLF)-like MAL and related proteins for vesicle trafficking transmembrane, domain-containing member 5
HCC: hepatocellular carcinoma
CKLF: chemokine-like factor
HRP: horseradish peroxidase
MOI: multiplicity of infection
MMP: matrix metalloproteinase
CCK-8: cell counting kit-8
AFP: α-fetoprotein
ALT: alanine transaminase
GGT: gamma-glutamyl transferase
EGFR: epidermal growth factor receptor
ERK: extracellular signal-regulated kinase
