# Supplementary material for: CMTM5 is downregulated and suppresses tumour growth in hepatocellular carcinoma through regulating PI3K-AKT signalling
Source: Cancer Cell Int. 2017 Nov 29;17:113. doi: 10.1186/s12935-017-0485-8 (PMC5707824; doi:10.1186/s12935-017-0485-8)
Supplement: Supplementary file 1 — Additional file 1: Table S1. Primers used in this study. [file 12935_2017_485_MOESM1_ESM.docx]

Table S1. Primers used in this study.

| Primer | Forward | Reverse |
| --- | --- | --- |
| CMTM5 | GGAGGACCACATCCGCTAGAT | CCAGGGAGTGGAAGCAGAT |
| AKT | AGCGACGTGGCTATTGTGAAG | GCCATCATTCTTGAGGAGGAAGT |
| PI3K | TGGACGGCGAAGTAAAGCATT | AGTGTGACATTGAGGGAGTCG |
| P21 | GGGATCTCTATGTCGGCATGT | CACACCTTCCTATCAGCATGAG |
| cyclin D1 | GCTGCGAAGTGGAAACCATC | CCTCCTTCTGCACACATTTGAA |
| cyclin E | GCCAGCCTTGGGACAATAATG | CTTGCACGTTGAGTTTGGGT |
| Bcl2 | GGTGGGGTCATGTGTGTGG | CGGTTCAGGTACTCAGTCATCC |
| Bax | CCCGAGAGGTCTTTTTCCGAG | CCAGCCCATGATGGTTCTGAT |
| Bad | CCCAGAGTTTGAGCCGAGTG | CCCATCCCTTCGTCGTCCT |
| MMP2 | TACAGGATCATTGGCTACACACC | GGTCACATCGCTCCAGACT |
| MMP9 | TGTACCGCTATGGTTACACTCG | GGCAGGGACAGTTGCTTCT |
| GAPDH | CTGGGCTACACTGAGCACC | AAGTGGTCGTTGAGGGCAATG |
