# Supplementary figures and images for: CMTM5 is downregulated and suppresses tumour growth in hepatocellular carcinoma through regulating PI3K-AKT signalling
Source: Cancer Cell Int. 2017 Nov 29;17:113. doi: 10.1186/s12935-017-0485-8 (PMC5707824; doi:10.1186/s12935-017-0485-8)

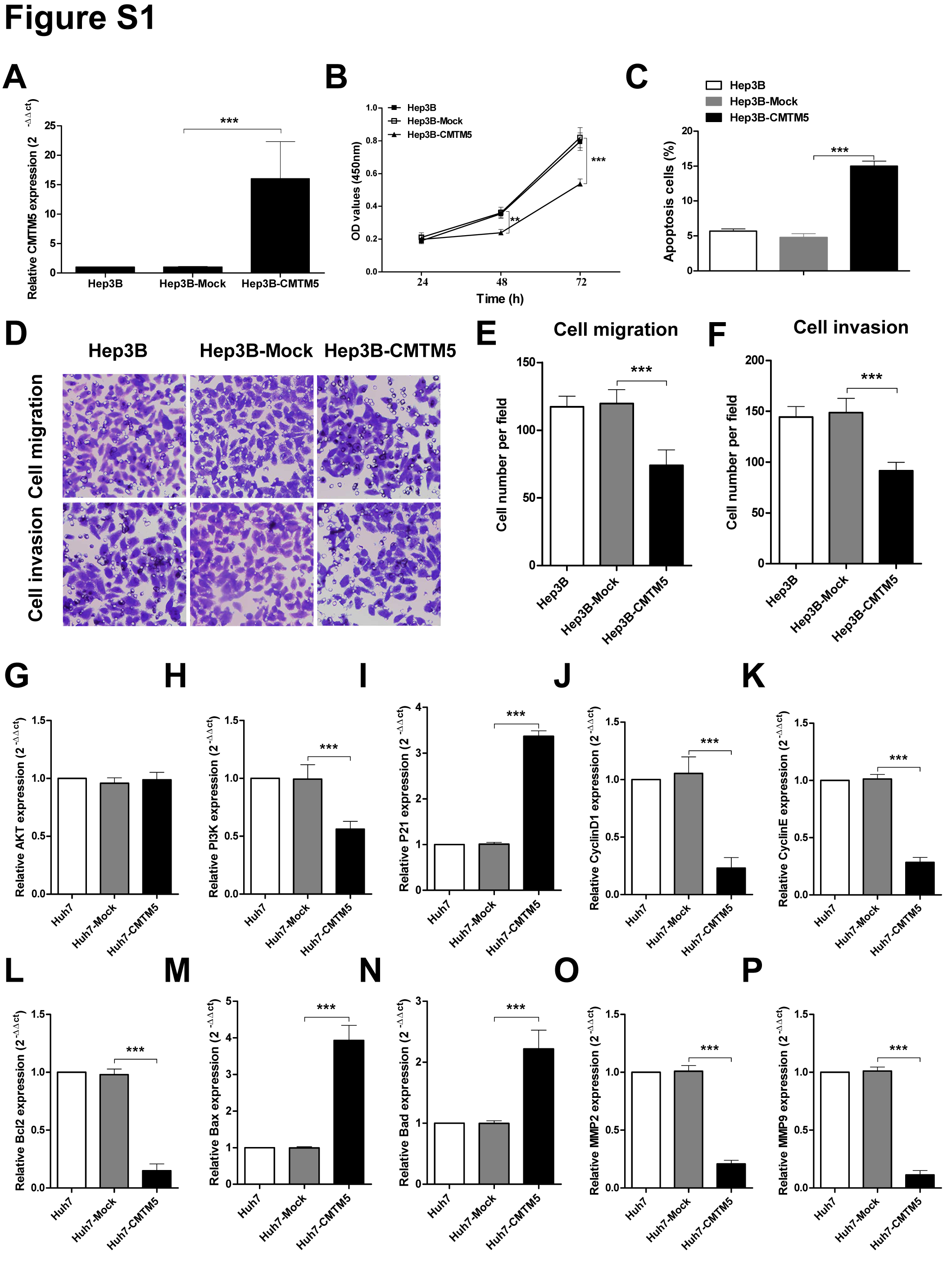

Supplement: Supplementary file 2 — Additional file 2: Figure S1. Overexpression of CMTM5 inhibits HCC cell growth and metastasis in Hep3B cells. (A) Lentivirus vector pLenti6.3-CMTM5-IRES-EGFP and the mock control lentivirus were transfected into Hep3B cells. Transfection efficiency was confirmed by qRT-PCR and western blotting. (B) Cell growth was assessed by time course CCK-8 assay. (C) Cell apoptosis was determined by flow cytomety using the Annexin V/PI staining. (D–F) Cell metastatic and invasion was assessed by Transwell assay. (G–P) The mRNA levels of PI3K/Akt pathway, including AKT, PI3K p85, p21, CyclinD1, CyclinE, Bcl2, Bax, Bad, MMP2 and MMP9 in CMTM5 overexpressed Huh7 cells were examined by real-time quantitative RT-PCR. Data are shown as mean ± SD from three independent experiments. *P < 0.05, **P < 0.01, ***P < 0.001, compared with Huh7-Mock group (One-Way ANOVA with Dunnett’s test). [file 12935_2017_485_MOESM2_ESM.tif]
